# Effects of self-care intervention using a mobile instant messenger on hemodialysis patient’s knowledge, self-efficacy, self-care behavior and physiological index

**DOI:** 10.7586/jkbns.24.004

**Published:** 2024-05-28

**Authors:** Yu Kyung Shin, Mi Young Kim

**Affiliations:** 1 College of Nursing, Hanyang University, Seoul, Korea; 2 Seoul National University Hospital, Seoul, Korea; 3 College of Nursing, Hanyang University, Seoul, Korea

**Keywords:** Renal dialysis, Self-efficacy, Self-care, Potassium, Weight gain

## Abstract

**Purpose:**

The purpose of this study was to examine the effects of self-care intervention using a mobile instant messenger on hemodialysis patient`s knowledge, self-efficacy, self-care behavior and physiological indices.

**Methods:**

A non-equivalent control group pretest-posttest design was used. The participants consisted of 38 patients who had a regular hemodialysis of Seoul National University Hospital. They were assigned to one of two groups; an experimental group (n = 19) that had self-care intervention for 8 weeks and a control groups (n = 19) that had routine hemodialysis treatment. The data collection was conducted from September 5, 2022 to October 29, 2022. The date were analyzed by Chi-square test, Independent t-test, and Fisher`s exact test using SPSS Win 29 program.

**Results:**

There was a significant difference in self-efficacy (t = 3.42, *p* = .002) between experimental and control groups. There was no significant differences between the two groups in knowledge (t = 0.80, *p* = .428), self-care behavior (t = 0.09, *p* = .929), potassium (t = -0.82 *p* = .416), phosphorus (t = -0.03, *p* = .974), weight gain (t = 0.16, *p* = .867).

**Conclusion:**

Based on above results, it was verified that self-care intervention in this study was an effective indicator in improving the self-efficacy. There is a requirement to formulate comprehensive interventions capable of enhancing various indicators.

## INTRODUCTION

### 1. Significance of the study

The incidence of end-stage renal disease (ESRD) has continued to increase. In 2022, 134,826 people were reported to have ESRD in South Korea, and the proportion of patients undergoing hemodialysis is increasing every year [1]. ESRD is a disease in which kidney function is impaired owing to a range of etiologies, including chronic glomerulonephritis, and the glomerular filtration rate is irreversibly reduced. Renal replacement therapy is necessary when the filtration rate decreases to 10% of the patients normal range [2]. Among ESRD patients, the proportion of those receiving hemodialysis is high (83.6 %). These patients are required to visit the hospital 2-3 times a week for 3-4 hours of dialysis during each visit [1]. Despite receiving continuous dialysis treatment, patients experience physical and emotional symptoms, such as fatigue, lack of energy, dry skin, itching, sleep disorders, and anxiety owing to insufficient excretion of water, salt, and waste products [3]. The hospitalization rate in Korea is increasing owing to the comorbidities of dialysis patients and the side effects of treatment, rising from 76.2% per 1,000 people in 2013 to 292.3% by 2022 [1]. In addition, the mortality rate of dialysis patients is increasing; rubbing shoulders with the rates of cardiovascular diseases and infections [2]. Patients with chronic illnesses, including hemodialysis patients, show low adherence to self-care behaviors, such as engaging in regular physical activity, taking prescribed medications, and maintaining a proper diet [4]. Therefore, self-care is vital for disease management because it helps reduce chronic physical and mental disease complications [5].

In general, people with chronic diseases need to actively and effectively perform self-care to manage their illness [4]; hemodialysis patients need to engage in self-care to maintain their life, health, and well-being. It is a personal endeavor and practical act that an individual needs to take to reduce disease progression, prevent complications, and maintain overall health [6]. One may think that their disease is managed with hemodialysis alone, and although the treatment relieves symptoms of uremia and reduces the risk of organ failure, it only assumes the excretory function of the kidneys. Therefore, other self-care measures are important to prevent side effects and complications [7]. It is especially important for hemodialysis patients to pay attention to their health and proactively engage in self-care to manage their illness and achieve an optimal quality of life [8].

In this regard, self-care for hemodialysis patients refers to what patients do to maintain their health. These include managing arteriovenous fistulas, taking medications, following dialysis schedules, and managing diets and fluids [9]. Previous studies have shown that self-care in hemodialysis patients is significantly correlated with knowledge and self-efficacy [10], and that knowledge of hemodialysis and self-care behaviorare positively correlated [11]. Topics related to hemodialysis include kidney function, blood vessel management, medication management, dialysis, exercise, and daily routines [12]. Improving treatment knowledge is important to ensure effective disease management [13]. Prior research has shown that self-care behavior improves in hemodialysis patients when they acquire accurate knowledge about dialysis and disease managementand build self-efficacy [12]. A study on older hemodialysis patients found that increased self-efficacy encourages them to stay on life support and prevents further deterioration in their health [14]. Other studies have reported that higher self-efficacy is associated with better performance of self-care behaviors [9].

Although self-care showed a significant impact on hemodialysis patients overall, physiological indicators provided differing results depending on the type and duration of self-care interventions. In a study that investigated personalized nutrition education, hemodialysis dietary knowledge, self-care behavior, and self-efficacy significantly increased after patients received education. However, physiological indicators did not show significant differences at 4 or 12 weeks after the intervention [15]. Another study that implemented a video program found that only potassium levels were significantly affected, whereas phosphorus levels and weight gain were not [16]. These results suggest that there is a need to replicate studies examining the physiological indicators of self-care in hemodialysis patients.

With the recent advances in science and technology, mobile applications have become widely used in healthcare [12]. Interventions using mobile applications are increasing healthcare access and providing access to readily available information as well as convenient and recipient-centered services [17]. In addition, they promote communication with medical staff, reduce treatment costs, and strengthen patients’ motivation to take therapeutic action [18]. Mobile applications can be seen as an efficient medium that transcends physical constraints such as time and place. A mobile application-based self-help program study with hemodialysis patients showed that a dietary program intervention using text messaging had a significant effect on physiological parameters [19]. However, there have been reports of inconveniences, such as “login errors,” “linkage errors,” “update errors,” “execution errors,” “‘slowness,” and “poor management” when using health-related mobile applications, indicating that the smooth management of applications requires considerable time and resources [20]. In terms of usability and accessibility, it is worth examining whether interventions communicating through mobile-based text messages, even if they are not a mobile application-level program, are helpful in providing long-term self-care for hemodialysis patients. This could help patients undergoing hemodialysis perform self-management.

This study aimed to provide evidence on effective nursing interventions for hemodialysis patients by determining the effects of an 8-week self-care intervention using the mobile instant messenger application, KakaoTalk, on hemodialysis patients’ knowledge, self-efficacy, self-care behavior, and physiological indices.

### 2. Study hypotheses

The hypotheses of this study were as follows.

1) The experimental group that received the self-care intervention will have more hemodialysis knowledge than the control group.

2) The experimental group that received the self-care intervention will have a stronger sense of self-efficacy than the control group.

3) The experimental group that received the self-care intervention will have higher self-care behavior scores than the control group.

4) The experimental group that received the self-care intervention will have lower blood potassium levels than the control group.

5) The experimental group that received the self-care intervention will have lower blood phosphorus levels than the control group.

6) The experimental group that received the self-care intervention will have less weight gain than the control group.

## METHODS

### 1. Study design

This was a quasi-experimental study with a non-equivalent control group and a pre-post design that aimed to determine the effect of a self-care intervention centered on communication via amobile instant messenger on hemodialysis knowledge, self-efficacy, self-care behavior, and physiological indices in hemodialysis patients. A previous study, with similar variables in hemodialysis patients, did not implement the same intervention as this study and found significant pre- to post-intervention effects on some items, but not on others, when comparing the 4- to 12-week marks after a 4-week intervention [15]. Based on this finding, we extended the duration of the intervention to once a week for eight weeks.

Before the intervention was implemented, the experimental and control groups received an education session which lasted 30 minutes using a booklet prepared by the researcher. Subsequently, a preliminary survey was administered, in which the survey items measured hemodialysis-related knowledge, self-efficacy, self-care behavior, and physiological indicators. The experimental group then received a self-care intervention once a week for eight weeks, while the control group received routine care. A follow-up survey measuring hemodialysis-related knowledge, self-efficacy, self-care behavior, and physiological indicators was conducted at week nine (Table 1).

### 2. Study participants

The participants in this study were selected among dialysis patients receiving hemodialysis more than twice a week at the hemodialysis unit of Seoul National University Hospital in Seoul from July 1 to August 13, 2022. These patients had been on dialysis for more than three months, which is the period for receiving a disability rating from the National Health Insurance Service [21]. Based on previous studies, the required sample size was calculated using the G*power 3.1.9.7 program with an effect size of .83, number of groups of 2, significance level of .05, and power of 0.7 [22]. Using these values, the experimental and control groups comprised 19 participants each. However, considering a dropout rate of 10%, 42 participants were selected, with 21 in the experimental and control groups. Participants were required to contact the researcher via a mobile-based text message once a week and were excluded withdrawn from the study if they could not be contacted more than twice.

The inclusion criteria were as follows: patients aged 18 years or older, those who had been on hemodialysis for at least three months and were receiving outpatient maintenance dialysis, those who were able to read and communicate in Korean, those who owned a cell phone with messenger services and were able to send and receive messages, those who were able to perform independent self-care, and those who understood the purpose of the study and agreed to participate. Since chronic kidney disease has diverse etiologies, other comorbidities were not excluded. Among patients who started dialysis for more than three months, those who underwent dialysis more than twice a week were excluded. Patients on a combination of hemodialysis and peritoneal dialysis and those hospitalized during the study period were also excluded.

A total of 42 participants expressed willingness to participate in the study. The patients were divided into experimental and control groups with 21 patients in each group. Subsequently, two participants in the control group withdrew due to kidney transplantation during the intervention, one participant in the experimental group withdrew because they refused to participate in the study just prior to the intervention, and four participants withdrew owing to a change in condition one week after the intervention, finally resulting in a total of 38 participants.

### 3. Study tools

#### 1) Participant characteristics

The general characteristics of the participants were collected using a questionnaire that included sex, age, education, religion, marital status, occupation, and type of insurance. Disease-related characteristics were collected using a questionnaire that included regular outpatient visits, methods of collecting dialysis-related information, primary caregiver, who paid for treatment, duration of hemodialysis, and whether the patient had been hospitalized since hemodialysis.

#### 2) Hemodialysis knowledge

Hemodialysis knowledge was measured using a 20-item tool developed by Hong [23] and modified by Song [24]. It measures the level of knowledge of hemodialysis and consists of 20 questions in seven domains: normal kidney function and disease characteristics, hemodialysis, medication, diet, exercise, daily life, complications, and follow-up and treatment. Correct answers were scored 1 point, whereas incorrect answers were scored 0 points. The scores ranged from 0 to 20, with higher scores indicating greater knowledge of hemodialysis. Reliability was Cronbach’s α .76 in the study by Song [24], and .75 for KR-20 pre- and .81 post-test in this study.

#### 3) Self-efficacy

Self-efficacy was measured using a 10-item tool developed by Kim [25] for hemodialysis patients and modified by Choi and Lee [26]. Each item is rated from 1 to 4 on a 4-point Likert scale ranging from 1 (disagree) to 4 (strongly agree). Higher scores indicate stronger sense of self-efficacy. Reliability was a Cronbach's α of .77 in Choi and Lee’s [26] study and .77 pre- and .87 post-test in this study.

#### 4) Self-care behavior

Self-care behavior was measured using the hemodialysis patient self-care behavior measurement tool developed by Song et al. [27] and modified by Cho and Choe [22]. It consists of 35 questions on a 5-point Likert scale, with six questions on diet, six on blood vessel management, four on exercise and rest, two on medication use, three on blood pressure and weight management, 11 on physical management, and three on social life. Each question is scored on a scale from 1 (not doing well at all) to 5 (doing very well). The scores range from 35 to 175, with higher scores indicating better self-care behaviors. Reliability was Cronbach’s α .87 in the study by Cho and Choe [22] and .92 pre- and .93 post-test in this study.

#### 5) Physiological indicators

Physiological indicators refer to the measured values of blood potassium and phosphorus levels and weight gain in patients undergoing hemodialysis [22]. Blood potassium and phosphorus levels were collected from electronic medical records. Body weight was measured using a CAS medical scale (WCS-200, CAS, Seoul, Korea) immediately before and after dialysis by the researcher and a nurse from the hemodialysis unit at Seoul National University Hospital, with the patients wearing only a hospital gown. The weight gain was calculated by subtracting the weight measured immediately before dialysis from that measured immediately after dialysis. This was measured as the average value of the week before intervention and the average value of the week immediately after the intervention.

### 4. Study procedures

#### 1) Booklet production

The researchers modified and supplemented the Korean Society of Nephrology’s evidence-based guidelines for appropriate hemodialysis care and the “Chronic Kidney Disease at a Glance” booklet, validated the content with a group of experts, and conducted a pre-education session using the booklet. The booklet included information on chronic kidney disease, diets, medications, and exercise management. Content validity was confirmed by a group of experts, including a nephrologist, two nurses with more than 10 years of experience in hemodialysis units, and a nursing professor.

#### 2) Common education session

A common education session was conducted between August 15 and August 25, 2022. According to a previous study, as the age of hemodialysis patients increased, their literacy level decreased; the lower the literacy level, the lower the level of knowledge about the disease [10]. Based on this, the ages of the participants in this study ranged from under 40 to over 65 years. Considering that this could affect the level of knowledge, a common education session was conducted. The session was held in a conference room in the dialysis unit, with groups of 6-9 people in both the experimental and control groups. Participants gathered in the conference room approximately an hour earlier on one of the dialysis days between August 15 and 25 for a single 30-minute session using the booklet.

#### 3) Preliminary survey

As part of a preliminary survey, questionnaires on general and disease-related characteristics, hemodialysis knowledge, self-efficacy, and self-care behavior were distributed to the experimental and control groups immediately after the education session on the day of dialysis. Physiological indicators of blood potassium and phosphorus were measured using the results of a routine blood test in August, the month of the preliminary survey. Weight gain was measured as the average of the week prior to the start of the study, as patients showed more weight gain on Mondays and Tuesday than on other days.

#### 4) Self-care interventions using mobile instant messengers

The experimental treatment with self-care intervention was conducted from September 5, 2022, to October 29, 2022. The patients were divided into the experimental and control groups by the researcher. The self-care intervention provided to the experimental group was conducted through one-on-one KakaoTalk chats for approximately 40 minutes, once a week for eight weeks.

After completing the preliminary questionnaire and before conducting the study, we met the experimental group in person in the dialysis room, provided them with the researcher’s KakaoTalk ID, and added them as friends. We then explained that the study would be conducted through the KakaoTalk chat. During the consultation, participants were asked to send photos or text messages about their meals and exercise routines during non-dialysis days on the day they ate and performed, respectively. If it was impossible to send it on the day of consultation, we explained that a week’s worth of meals and exercise routines could be sent simultaneously. Each participant in the experimental group was contacted during the first week of September to schedule the date and time of the first consultation. Starting from the second consultation, subsequent consultations were scheduled at the end of each consultation using KakaoTalk on the day the experimental group participants had time that week. The self-care interventions used in this study are shown in Table 2.

##### (1) Goal setting

First, the appropriate goals were set. Goal setting was based on an analysis of stress in hemodialysis patients [28]. The intervention was conducted by selecting subgoals for each participant based on the following three major objectives:

§ Ensure that patients who respond well to their current treatment remain on the treatment without any changes in their condition.

§ Ensure that patients who gain too much or too little weight maintain weight gain within 4% of their target weight.

§ Adjust the diet for patients with electrolyte imbalance issues.

##### (2) Action and evaluation

We went through a process of having the participants and nurses agree on small goals, such as “I’ve been eating too much bread this week. I think 2-3 times a day is a bit much, so I'll try cutting back to once a day.” or “I didn’t realize I shouldn’t eat three different types of fruit. I am going to cut it down to one a day.” During the consultation held the following week, we received photos and texts of meals and exercise routines to confirm whether the goals were achieved.

Toward the consultation’s end, the participants were encouraged to keep up with their progress. The next consultation was scheduled before the conclusion of the session. We explained to them that if they had any questions outside of counseling time, we were happy to answer them and encouraged them to send their questions through KakaoTalk. The intervention was implemented once a week for eight weeks (Table 2).

The control group received the same dialysis treatment without any treatment other than the prior education.

#### 5) Follow-up survey

From November 1 to November 8, 2022, we met the experimental and control groups in person in the hemodialysis room and administered questionnaires on hemodialysis-related knowledge, self-efficacy, and self-care behavior. Physiological indices, such as blood potassium and phosphorus, were measured using the test results from November, the month of the follow-up survey, and monthly blood tests. Weight gain was measured as the average weight of the week after the end of the intervention, as the increase in weight was higher on Mondays and Tuesdaythan on other days.

### 5. Data analysis

The collected data were analyzed using IBM SPSS Statistics software (version 29.0; IBM., Armonk, NY, USA). Frequency analysis and descriptive statistics were used to determine the general and disease-related characteristics of the experimental and control groups. The preliminary homogeneity test according to the general and disease-related characteristics of the experimental and control groups was verified using the chi-squared test, independent t-test, and Fisher’s exact test. Knowledge of hemodialysis in the experimental and control group’s self-efficacy, self-care behavior, physiological indices, and questionnaire scores were analyzed using mean and standard deviation. Knowledge of hemodialysis before and after the self-care intervention in the experimental and control groups and differences in self-efficacy and self-care behavior were verified using an independent t-test. The Cronbach’s alpha, coefficient, and KR-20 were calculated to validate the reliability of the tools used in this study.

### 6. Ethical considerations

This study was conducted after review and approval from the IRB of Seoul National University Hospital (IRB No. H-2205-010-1320). Before collecting the data, we explained the consent form to the participants and informed them that their participation in the study was voluntary. Written informed consent was obtained and incorporated into the study, explaining that there were no risks or penalties to the participants, and that they could withdraw at any time during the study. One week after the program ended all the participants received a small prize.

## RESULTS

### 1. Homogeneity test for general and disease-related characteristics

There were 11 males (57.9%) in the experimental group and 11 females (57.9%) in the control group. The average age of the experimental group was 52.84 ± 11.69 years and for the control group 61.58 ± 13.41 years. The sample comprised 10 college graduates (52.6%) in the experimental group and nine high school graduates (47.4%) in the control group. Regarding religion, six people (31.6%) were Christians and 6 (31.6%) were Catholics in the experimental group, whereas 8 (42.1%) were Atheism in the control group. In terms of marital status, 10 (52.6%) and 13 (68.4%) were married in the experimental and control groups, respectively. Twelve (63.2%) participants in the experimental group and 16 (84.2%) in the control group were unemployed. The homogeneity test of the general characteristics of the experimental and control groups revealed no significant differences, confirming that the groups were homogeneous (Table 3).

Thirteen (68.4%) and 12 (63.2%) patients regularly visited a nephrologist in the experimental and control groups, respectively. The sources of dialysis information from medical staff included 17 providers (73.9%) in the experimental group and 14 providers (63.6%) in the control group. In terms of primary caregivers of dialysis patients, 8 (42.1%) participants in the experimental group marked their spouses as their primary caregivers, while 11 (57.9%) in the control group marked “other” as their primary caregiver. The treatment cost was borne by 11 (57.9%) in the experimental group and 8 (42.1%) in the control group. The duration of dialysis was between six and then years for eight participants (42.1%) in the experimental group and 7 (36.8%) in the control group. Regarding hospitalizations other than for dialysis, 11 (57.9%) patients in the experimental group and 16 (84.2%) in the control group were hospitalized. A homogeneity test of the disease-related characteristics of the experimental and control groups confirmed that they were homogeneous with no significant differences (Table 3).

### 2. Hypothesis test

The results of the hypothesis tests for the dependent variables for the experimental and control groups who received the 8-week mobile instant messaging self-care intervention are shown in Table 4.

When measuring the knowledge of hemodialysis between the experimental and control groups, there was no statistically significant difference (t = 0.80, *p* = .428), with the experimental group scoring 18.16 ± 1.01 and the control group scoring 17.84 ± 1.38. Therefore, Hypothesis 1, the experimental group receiving the self-care intervention would have more hemodialysis knowledge than the control group, was rejected.The post-intervention self-efficacy scores of the experimental and control groups were 33.05 ± 4.87 for the experimental group and 27.37 ± 5.35 for the control group, showing a significant difference (t = 3.42, *p* = .002). Therefore, Hypothesis 2, the experimental group receiving the self-care intervention will have a stronger sense of self-efficacy than the control group, was supported.The self-care behavior scores were 132.95 ± 16.30 for the experimental group and 132.32 ± 25.78 for the control group, which was not statistically significant (t = 0.09, *p* = .929). Thus, Hypothesis 3, the experimental group receiving the self-care intervention would have higher self-care behavior scores than the control group, was rejected.Among the physiological indicators of the experimental and control groups, blood potassium was 4.62 ± 0.78 for the experimental group and 4.80 ± 0.57 for the control group, which was not statistically significant (t = -0.82, *p* = .416). Therefore, Hypothesis 4, the experimental group receiving the self-care intervention will have lower blood potassium levels compared to the control group, was rejected. The blood phosphorus level was 4.70 ± 0.97 for the experimental group and 4.71 ± 0.97 for the control group, which was not statistically significant (t = -0.03, *p* = .974). Therefore, Hypothesis 5, the experimental group receiving the self-care intervention will have lower blood phosphorus levels compared to the control group, was rejected.The amount of weight gain was 1.92 ± 0.95 for the experimental group and 1.87 ± 0.64 for the control group, showing no statistically significant difference (t = 0.16, *p* = .867). Therefore, Hypothesis 6, the experimental group that received self-care intervention would have less weight gain compared to the control group, was rejected.

## DISCUSSION

This study was conducted to determine the impact of a self-care intervention using mobile instant messengers on hemodialysis knowledge, self-efficacy, self-care behavior, and physiologic indicators in hemodialysis patients after eight weeks to provide evidence for the use of mobile nursing interventions in these patients.

The results showed no significant difference in the experimental and control groups’ hemodialysis knowledge after the self-care intervention.This differs from a study that found that nonadherent dialysis patients who received personalized nutrition education sessions had significantly more hemodialysis knowledge than those who did not [15].In this study, the knowledge scores prior to the education session were 91 in the experimental group and 89 in the control group on a scale of 100, which are higher than the scores of 79 and 86 after 12 weeks of intervention in the previous study [15], indicating that the knowledge level of the participants in this study was higher than after the intervention in the previous study. Given that the experimental and control groups in this study did not differ in terms of education and age, and that a study of chronically ill older patients showed significant differences across all age groups when nutrition education was implemented [29], it is likely that the pre-education session had an impact on both the experimental and control groups. This is consistent with research showing that dietary education leads to increased knowledge among hemodialysis patients [30]. Therefore, it is necessary to change how interventions are implemented to verify the effectiveness of providing information on hemodialysis through mobile instant messengers.

Participants in the experimental group had a significantly stronger sense of self-efficacy than those in the control group after the intervention. Our findings are similar to those of a study that developed a mobile application for hemodialysis patients [13] and another that examined the effectiveness of a self-management intervention for hemodialysis patients [31].The ability to access information through mobile devices at any time and place and receive relevant feedback and encouragement after performing health behaviors has been shown to increase self-efficacy and motivation to continue engaging in health behaviors [13].In this study, it appears that the process of checking weekly for any discomfort and consulting with researchers about diet and exercise increased participants’ self-efficacy. Given the effectiveness of mobile instant messengers in increasing self-efficacy among hemodialysis patients, it is likely that interventions that utilize them in clinical settings to maintain and increase self-efficacy will be beneficial.

There were no significant differences in self-care behavior between the experimental and control groups after the self-care intervention.Self-management using mobile applications had different findings compared to studies that found significant results in self-efficacy and self-care behavior [32], and from studies that evaluated self-management programs after they were implemented in hemodialysis patients [31]. In Hosseini’s study [32], a mobile application allowed patients to access hemodialysis-related information at any time. Another study applied a self-management program in which the experimental group was provided with four 100-minute face-to-face counseling sessions and small group discussions [31]. In our study, counseling was conducted using a mobile instant messenger for eight weeks. Mobile counseling was conducted once a week, and participants were encouraged to ask questions at any time. However, the number of times participants were physically contacted was small compared to the number of times the mobile application was used, or a face-to-face or small group counseling session through which individuals could readily access information whenever they needed it.In addition, it can be assumed that it was difficult to change long-standing habits in a short period of time. Given that Kim and Han [33] reported that eating habits were difficult to control in practice, it is likely that this study was similar. As mobile instant messengers can increase access to healthcare [17] and can be seen as an efficient way to obtain long-term services [18], it is necessary to study interventions that utilize mobile services to provide group counseling and adjust the number of consultations for hemodialysis patients to increase access to information and provide continuous feedback.

In this study, the implementation of a self-care intervention did not result in significant differences in blood potassium, blood phosphorus, and weight gain between dialysis sessions. Therefore, all hypotheses regarding the physiological indicators were rejected. In a study that analyzed changes in physiological indicators related to diet in hemodialysis patients over the first year, only weight gain was significant, whereas blood potassium and phosphorus were not [34], which is somewhat similar to the results of the present study. However, given that both pre- and post-test scores remained within the normal range, it is likely that the education session had a positive impact on the participants, which is similar to the findings of previous studies [35].

Blood potassium levels outside the normal range of 3.5-5.5 mEq/L can affect the heart muscle and lead to arrhythmias. It is also closely related to the patient’s dietary habits [36]. In addition, blood phosphorus levels often remain high in patients with end-stage renal failure, and high levels can lead to the calcification of blood vessels [37]. A study on personalized nutrition education in nonadherent hemodialysis patients did not show significant changes in physiological indicators such as potassium, phosphorus, or weight gain, which may have been because of the vegetarian nature of the Korean diet and the fact that most foods contain phosphorus, making it difficult for patients to control their diet [15]. This finding is similar to those of previous studies showing that although dialysis patients have knowledge of dietary restrictions, they have difficulty changing their dietary habits, requiring ongoing management of complications [35]. Furthermore, weight gain, blood glucose, and potassium levels are related to dietary habits and cannot be changed in the short term [38]. A dietary intervention using mobile instant messaging for hemodialysis patients showed a significant difference in weight gain at six months post-intervention, with a reduction in sodium intake [19]; however, research is needed on the duration of the intervention and how to sustain behavioral changes in participants.

Our study showed that self-care interventions using mobile instant messengers had a significant effect on hemodialysis patients’ self-efficacy but not on hemodialysis-related knowledge, self-care behavior, and physiological parameters. Interventions using mobile instant messengers are important because they provide a method of nursing intervention for hemodialysis patients. Further research is required to improve patient knowledge, self-care behavior, and physiological indicators.

## CONCLUSION

This quasi-experimental study with a non-equivalent control group, pre-post design attempted to determine the effect of a self-care intervention using mobile instant messengers on hemodialysis-related knowledge, self-efficacy, self-care behavior, and physiological indicators in hemodialysis patients and to propose a nursing intervention for hemodialysis patients. The results confirmed that self-care interventions using mobile instant messengers made a significant difference to the self-efficacy of hemodialysis patients. Since self-efficacy is a factor that influences behavior, it is necessary to explore interventions and the duration of these interventions that affect other outcomes, such as hemodialysis knowledge, self-care behavior, and physiological indices. Based on the findings of this study, we make the following suggestions. First, as the participants were recruited from only one institution, a follow-up study targeting participants from multiple institutions is needed. Second, ongoing and replicated research using different forms of virtual counseling is needed to provide effective self-care measures for patients undergoing hemodialysis.

## Figures and Tables

**Table 1.** Design of Self-care Intervention by Using Mobile Instant Message

	Common education (0 week)	Pre-test (0 week)	Self-care intervention (1-8 weeks)	Post-test (9 week)
Experimental group (E)	X1	E1	X2	E2
Control group (C)	X1	C1		C2

X1 = Provide a single 30-minute pre-training session for both experimental and control groups using a booklet; E1, C1 = Pretest of hemodialysis-related knowledge, self-efficacy, self-care behavior, and physiological index; X2 = Implement self-care intervention eight times, once weekly, over an 8-week period; E2, C2 = Posttest of hemodialysis-related knowledge, self-efficacy, self-care behavior, and physiological index.

**Table 2.** 8 Weeks of Self-care Intervention

Weeks	Themes	Contents
1	Orientation	- Introduction of program purpose and current status of the week
		- An overview of how to take and share photos of your diet and exercise on non-dialysis days and how to conduct research
		- Set a final goal after 8 weeks and detailed goals that can be improved by the 2nd week.
		- Questions and answers about diet and exercise during dialysis and daily life
		- Emotional support and setting a meeting date for next week.
2	Food bikes and protein and fat	- Share your current situation for the past week and any inconveniences during dialysis or inconveniences in your daily life.
		- Feedback after sending the group education about the 5 food bikes and protein and fat
		- Look at pictures of one week's diet and exercise and discuss with the patient areas for improvement and goals for the past week.
		- Set detailed goals that can be improved by week 3
		- Emotional support and setting a meeting date for next week.
3	Phosphorous and calcium	- Share your current situation for the past week and any inconveniences during dialysis or inconveniences in your daily life.
		- Feedback after transmission of group education about phosphorus and calcium
		- Look at pictures of one week's diet and exercise and discuss with the patient areas for improvement and goals for the past week.
		- Set detailed goals that can be improved by week 4
		- Emotional support and setting a meeting date for next week.
4	Sodium and potassium	- Share your current situation for the past week and any inconveniences during dialysis or inconveniences in your daily life.
		- Feedback after transmission of group education about Sodium and Potassium
		- Look at pictures of one week's diet and exercise and discuss with the patient areas for improvement and goals for the past week.
		- Set detailed goals that can be improved by week 5
		- Emotional support and setting a meeting date for next week.
5	Dry weight and nutritional status assessment	- Share your current situation for the past week and any inconveniences during dialysis or inconveniences in your daily life.
		- Feedback after transmission of group training on dry weight and nutritional status assessment
		- Look at pictures of one week's diet and exercise and discuss with the patient areas for improvement and goals for the past week.
		- Set detailed goals that can be improved by week 6
		- Emotional support and setting a meeting date for next week.
6	Eating out	- Share your current situation for the past week and any inconveniences during dialysis or inconveniences in your daily life.
		- Feedback after sending the group education about eating out
		- Look at pictures of one week's diet and exercise and discuss with the patient areas for improvement and goals for the past week.
		- Set detailed goals that can be improved by week 7
		- Emotional support and setting a meeting date for next week.
7	Exercise	- Share your current situation for the past week and any inconveniences during dialysis or inconveniences in your daily life.
		- Feedback after sending group training on exercise
		- Look at pictures of one week's diet and exercise and discuss with the patient areas for improvement and goals for the past week.
		- Set detailed goals that can be improved by week 8
		- Emotional support and setting a meeting date for next week.
8	Q & A	- Share your current situation for the past week and any inconveniences during dialysis or inconveniences in your daily life.
		- Q&A regarding group training content
		- Look at pictures of one week's diet and exercise and discuss with the patient areas for improvement and goals for the past week.
		- Emotional support and discussion about feelings and pros and cons of the 8-week program

**Table 3.** Homogeneity Test on General Characteristics of Participants (N = 38)

Variables	Categories	Exp. (n = 19)	Cont. (n = 19)	*t* or *χ*^2^	*p*
		n (%) or M ± SD	n (%) or M ± SD		
Sex	Male	11 (57.9)	8 (42.1)	0.94	.330
	Female	8 (42.1)	11 (57.9)		
Age (yr)	≤ 45	4 (21.1)	2 (10.5)	-2.05^†^	.906^†^
	46-55	8 (42.1)	3 (15.8)		
	56-65	4 (21.1)	6 (31.6)		
	≥ 66	3 (15.7)	8 (42.1)		
		52.84 ± 11.69	61.58 ± 13.41		
Education level	Middle school	0 (0.0)	2 (10.5)	3.11	.375
	High school	7 (36.9)	9 (47.4)		
	University	10 (52.6)	7 (36.8)		
	Graduate school	2 (10.5)	1 (5.3)		
Religion	Christian	6 (31.6)	4 (21.1)	4.40	.355
	Catholic	6 (31.6)	2 (10.5)		
	Buddhism	2 (10.5)	4 (21.1)		
	Atheism	4 (21.1)	8 (42.1)		
	Others	1 (5.2)	1 (5.2)		
Marital status	Single	9 (47.4)	3 (15.8)	6.39	.094
	Married	10 (52.6)	13 (68.4)		
	Divorce	0 (0.0)	1 (5.3)		
	Bereavement	0 (0.0)	2 (10.5)		
Occupation state	Yes	7 (36.8)	3 (15.8)	2.17	.269^‡^
	No	12 (63.2)	16 (84.2)		
Insurance status	Medical protection	4 (21.1)	1 (5.3)	2.07	.340^‡^
	Health insurance	15 (78.9)	18 (94.7)		
Visiting nephrologist regulary	Yes	13 (68.4)	12 (63.2)	0.11	.732
	No	6 (31.6)	7 (36.8)		
Dialysis information source^§^	Medical staff	17 (73.9)	14 (63.6)	1.57	.209
	Brochure or internet	6 (26.1)	4 (18.2)		
	Others	0 (0.0)	4 (18.2)		
Main guardian	Spouse	8 (42.1)	7 (36.8)	2.75	.252
	Parents	4 (21.1)	1 (5.3)		
	Others	7 (36.8)	11 (57.9)		
Who pays treatment costs	Myself	11 (57.9)	8 (42.1)	2.18	.335
	Spouse	3 (15.8)	7 (36.8)		
	Parents or child	5 (26.3)	4 (21.1)		
Dialysis period (yr)	≤ 5	7 (36.9)	3 (15.8)	3.61	.306
	6-10	8 (42.1)	7 (36.8)		
	11-15	2 (10.5)	5 (26.3)		
	≥ 16	2 (10.5)	4 (21.1)		
Hospitalization experience after dialysis	Yes	11 (57.9)	16 (84.2)	3.19	.151^‡^
	No	8 (42.1)	3 (15.8)		

Exp. = Experimental group; Cont. = Control group; M = Mean; SD = Standard deviation.

^†^Independent t-test, ^‡^Fisher`s exact test; ^§^Duplicate response.

**Table 4.** Comparison of Dependent Variables Score Between Experimental and Control Groups (N = 38)

Variables		Exp. (n = 19)	Cont. (n = 19)	*t*	*p*
		M ± SD	M ± SD		
Knowledge	Pre	18.21 ± 1.13	17.84 ± 1.34	0.80	.428
	Post	18.16 ± 1.01	17.84 ± 1.38		
Self-efficacy	Pre	30.74 ± 4.47	33.26 ± 4.96	3.42	.002
	Post	33.05 ± 4.87	27.37 ± 5.35		
Self-care behavior	Pre	129.05 ± 20.25	130.79 ± 20.36	0.09	.929
	Post	132.95 ± 16.30	132.32 ± 25.78		
Potassium (mEq/L)	Pre	4.70 ± 0.72	4.84 ± 1.03	-0.82	.416
	Post	4.62 ± 0.78	4.80 ± 0.57		
Phosphous (mg/dL)	Pre	4.63 ± 1.03	4.67 ± 0.69	-0.03	.974
	Post	4.70 ± 0.97	4.71 ± 0.97		
Weight gain (kg)	Pre	1.84 ± 0.85	1.86 ± 0.55	0.16	.867
	Post	1.92 ± 0.95	1.87 ± 0.64		

Exp. = Experimental group; Cont. = Control group; M = Mean; SD = Standard deviation; Pre = Pre-test; Post = Post-test.

## Data Availability

The data that support the findings of this study are available from the corresponding author upon reasonable request.
